# Genetically Predicted Obesity Causally Increased the Risk of Hypertension Disorders in Pregnancy

**DOI:** 10.3389/fcvm.2022.888982

**Published:** 2022-05-25

**Authors:** Wenting Wang, Jiang-Shan Tan, Lu Hua, Shengsong Zhu, Hongyun Lin, Yan Wu, Jinping Liu

**Affiliations:** ^1^Department of Cardiopulmonary Bypass, State Key Laboratory of Cardiovascular Disease, Fuwai Hospital, National Center for Cardiovascular Diseases, Chinese Academy of Medical Sciences and Peking Union Medical College, Beijing, China; ^2^Department of Anaesthesiology, Second Affiliated Hospital, Hainan Medical College, Haikou, Hainan, China; ^3^Center for Respiratory and Pulmonary Vascular Diseases, Department of Cardiology, Key Laboratory of Pulmonary Vascular Medicine, National Clinical Research Center of Cardiovascular Diseases, Fuwai Hospital, Chinese Academy of Medical Sciences and Peking Union Medical College, Beijing, China

**Keywords:** genetic susceptibility, obesity, hypertension disorders in pregnancy, two-sample, Mendelian randomization

## Abstract

**Aims:**

This study aimed to evaluate the causal association between obesity and hypertension disorders in pregnancy.

**Methods:**

Two-sample Mendelian randomization (MR) study was conducted based on the data obtained from the GIANT (*n* = 98,697 participants) consortium and FinnGen (*n* = 96,449 participants) consortium to determine the causal effect of obesity on the risk of hypertension disorders in pregnancy. Based on a genome-wide significance, 14 single-nucleotide polymorphisms (SNPs) associated with obesity-related databases were used as instrumental variables. The random-effects inverse-variance weighted (IVW) method was adopted as the main analysis with a supplemented sensitive analysis of the MR-Egger and weighted median approaches.

**Results:**

All three MR methods showed that genetically predicted obesity causally increased the risk of hypertension disorders in pregnancy. IVW analysis provided obesity as a risk factor for hypertension disorders in pregnancy with an odds ratio (OR) of 1.39 [95% confidence interval (CI) 1.21–1.59; *P* = 2.46 × 10^−6^]. Weighted median and MR Egger regression also showed directionally similar results [weighted median OR = 1.49 (95% CI, 1.24–1.79), *P* = 2.45 × 10^−5^; MR-Egger OR = 1.95 (95% CI, 1.35–2.82), *P* = 3.84 × 10^−3^]. No directional pleiotropic effects were found between obesity and hypertension disorders in pregnancy with both MR-Egger intercepts and funnel plots.

**Conclusions:**

Our findings provided directed evidence that obesity was causally associated with a higher risk of hypertension disorders in pregnancy. Taking measures to reduce the proportion of obesity may help reduce the incidence of hypertension disorders in pregnancy.

## Introduction

Hypertension disorders in pregnancy (HDP) are defined as elevated office blood pressure ≥140/90 mmHg during the pregnancy (1), which is the most common complication of gestation, affecting up to 10% of pregnant women all over the world (2). HDP is also the major cause of morbidity and mortality in maternal, fetal, and neonatal and is associated with an increased risk of multiple organ failure, placental disruption, disseminated intravascular coagulation, major cardiovascular events, and death for the mother, and a higher risk of the fetus and newborn in intrauterine growth retardation, intrauterine death, stillborn, premature delivery, neonatal death (3, 4). Therefore, it is of practical significance to explore modifiable risk factors of HDP and make early predictions such that interventions can be carried out in advance to reduce the adverse effect brought by HDP. Many studies have shown that there is an observational correlation between obesity and the risk of HDP (5, 6). Since previous studies have only described that obesity can increase the risk of HDP, but how much risk can be increased, there is no relevant research data. Meanwhile, these observational studies were susceptible to reverse causality and confounding risk factors. As a consequence, whether the observed associations between obesity and HDP are causal is unclear.

**Figure 1:**
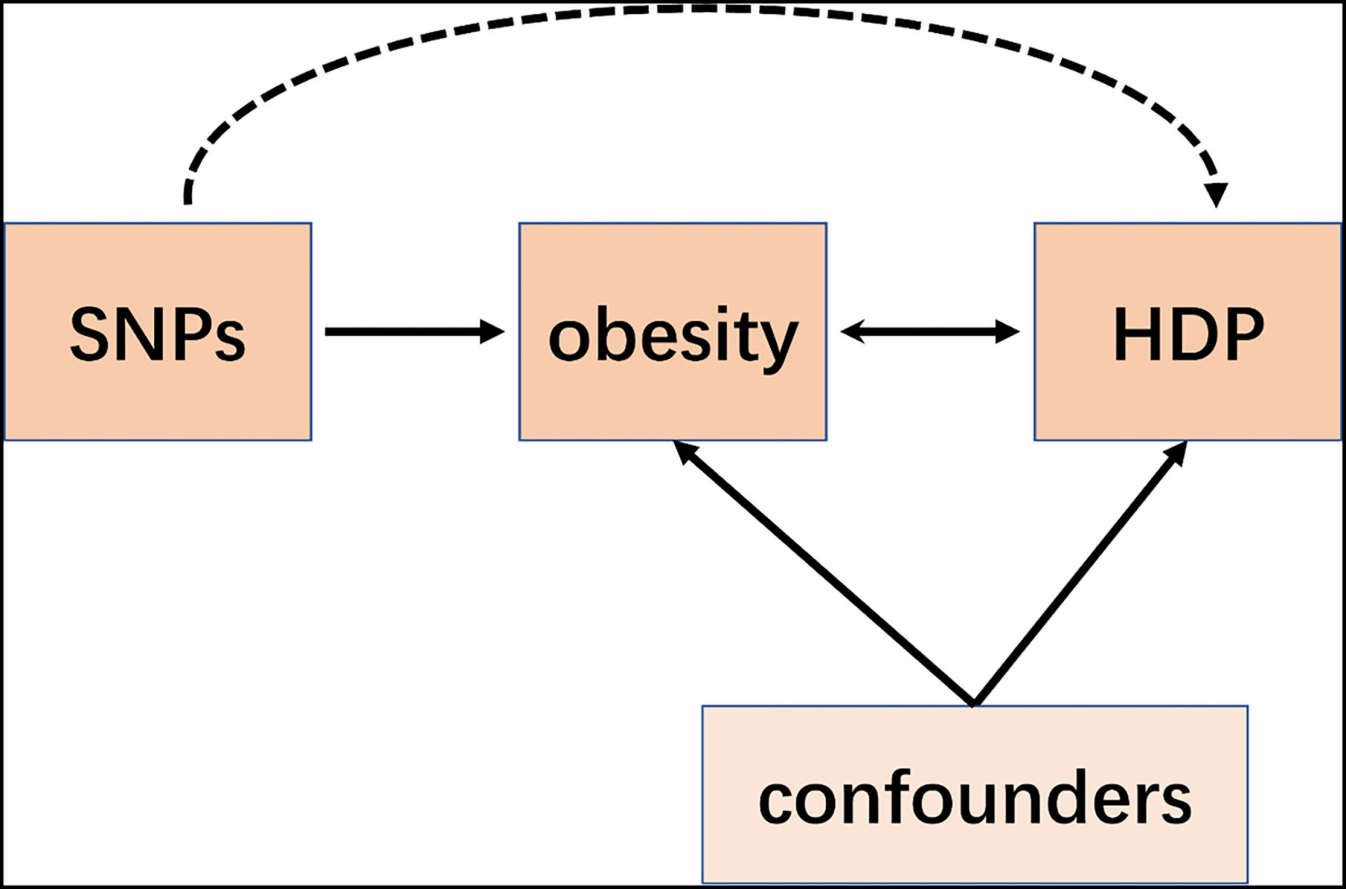
Schematic representation of an MR analysis. We selected SNPs which associated with obesity and the corresponding effect for these SNPs was estimated based on the risk of HDP obtained from a large cohort of the European population.

Mendelian Randomization (MR) is a recent emerging technique that applies single-nucleotide polymorphisms (SNPs) associated with risk factors as instrumental variables (IVs) to determine whether the observational association between a risk factor and a specific disease is causal (7–9). Although a MR study was performed retrospectively, it is similar to prospective randomized controlled trials (RCT) conceptually (Figure 1). Since all the germline hereditary genetic variants start with the formation of a zygote as a result of the fertilization of an oocyte, which occurs before the disease onset. Therefore, the potential bias of confounding or non-differential measurement error can be avoided in a MR study (10).

In this study, we aimed to explore the causal association between obesity and the risk of HDP from published genome-wide association studies (GWAS) correlation data by using two-sample MR analysis.

## Materials and Methods

### Study Design

In this study, we performed a two-sample MR (11, 12) to investigate the causal association between obesity with increased risk of HDP. Thus, our study does not require further sanction since the published studies were used for the data extraction, which has been approved by their respective institutional review committee and the informed consent of the participants has been obtained in their original research.

### Data Sources

#### Study Exposure: Obesity

Summary-level genetics were acquired from published GWAS, the Genetic Investigation of Anthropometric Traits (GIANT) consortium, which included 32,858 obesity patients and 65,839 controls from Europe. The IVs we selected satisfy the following criteria: (1) The obesity-associated SNPs with a genome-wide significance (*P* < 5 × 10^−8^). (2) For avoiding bias caused by strong linkage disequilibrium among SNPs, the linkage disequilibrium of obesity-associated SNPs must satisfy the *r*^2^ <0.001 and window size = 10,000 kb. (3) We selected the SNPs with F statistic >10 to avoid the effect of weak IVs. F statistic = (β/SE)^2^. Totally, 14 obesity-associated SNPs were obtained as IVs, and the details of IVs were presented in Table 1 (13, 14).

**Table 1:** List of genetic instruments for obesity and log odds ratios of hypertension disorders in pregnancy risk by each instrumental SNP (GWAS significance with *P* < 5× 10^−8^ and linkage disequilibrium threshold with *R*^2^ < 0.001).

**No**	**SNP**	**Gene**	**Chr**	**EA**	**OA**	**EAF.obesity**	**EAF.HDP**	**Obesity β(SE)**	**HDP β(SE)**
1	rs987237	*TFAP2B*	6	A	G	0.10	0.79	0.13	−0.06
2	rs13393304	_	2	G	A	0.89	0.84	0.18	0.06
3	rs13130484	_	4	C	T	0.42	0.52	0.11	−0.03
4	rs7138803	_	12	A	G	0.44	0.38	0.08	0.04
5	rs527248	_	1	A	G	0.26	0.82	0.11	−0.06
6	rs887912	_	2	T	C	0.62	0.25	−0.08	−0.01
7	rs10182181	_	2	A	G	0.50	0.58	0.07	0.03
8	rs9816226	_	3	T	A	0.85	0.85	0.11	0.06
9	rs2307111	*POC5*	5	T	C	0.43	0.58	−0.07	−0.02
10	rs4929923	*TRIM66*	11	T	C	0.73	0.34	0.08	−0.04
11	rs8028313	*MAP2K5*	15	G	C	0.78	0.22	−0.10	−0.06
12	rs11075989	*FTO*	16	C	T	0.44	0.41	0.21	−0.09
13	rs523288	_	18	T	A	0.62	0.29	0.13	0.03
14	rs29939	_	19	A	G	0.22	0.67	0.07	0.01

*Chr. indicates chromosome; EA, effect allele; OA, other allele; EAF, effect allele frequency. A total of 14 genetic instruments were included in the present MR analysis, located in four genes (POC5, TRIM66, MAP2K5, and FTO) and 12 chromosomes (1, 2, 3, 4, 5, 6, 11, 12, 15, 16, 18, and 19) with an effect allele frequency (EAF) of 0.1–0.9 in SNPs of obesity and 0.2–0.9 in SNPs of hypertension disorders in pregnancy*.

#### Study Outcome: Pregnancy Hypertension

We obtained the GWAS summary data of HDP from the FinnGen project, which was acquirable at https://gwas.mrcieu.ac.uk/datasets/finn-a-HYPTENSPREG/. Our project contained 3,363 pregnancy hypertension cases and 93,136 controls. Totally, 14 SNPs were correspondingly found in the database. Consequently, the final MR analysis was finished based on all the SNPs found in the exposure.

### Statistical Analysis

Due to no individual-level GWAS data available in our study, we leveraged two-sample MR analyses, as mentioned previously (15, 16), to assess the causal association between obesity and HDP. Horizontal pleiotropy is that the outcome may be exposed by other pathways but not only the exposure, which violates the assumption of MR and can bias causal estimates. To prevent this, we use three different analysis methods in the present MR analysis. Each statistical approach conducted is based on different horizontal pleiotropy models. To make our results more reliable, we compared the consistency of all results in three different statistical approaches (17), including inverse-variance weighted (IVW) method, MR-Egger and weighted median MR methods. The “mRnd” tool was used to calculate the power of the present MR analysis. All of the statistical analysis was finished based on the MR software packages version 0.5.6 and R version 4.1.2 (2021-11-15) (18).

## Results

### Genetic Instruments of Obesity

**Figure 2:**
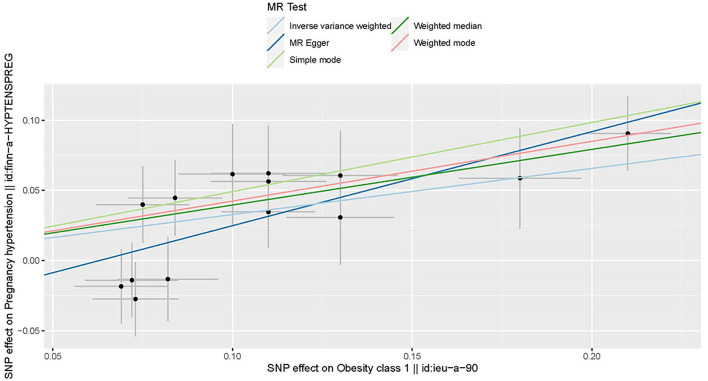
Scatter plot to visualize the causal effect of obesity on the risk of hypertension disorders in pregnancy (HDP). Each black point representing an SNP is plotted in relation to the effect size of the SNP on obesity (x-axis) and on the risk of HDP (y-axis) with corresponding standard error bars. The slope of each line corresponds to the causal estimate using different MR methods. IVW indicates inverse-variance weighted; and MR, Mendelian randomization.

**Figure 3:**
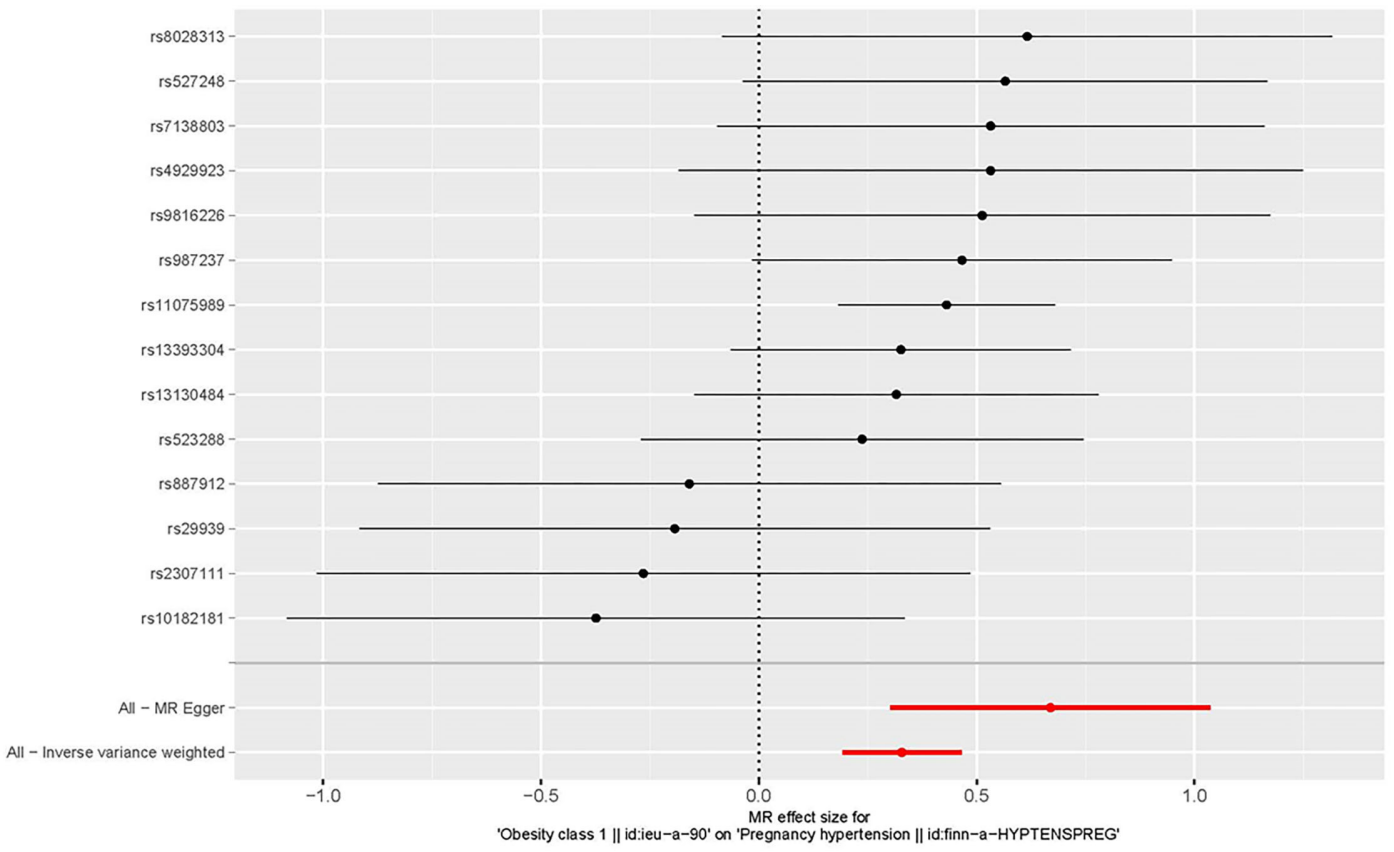
Forest plot to visualize the causal effect of every single SNP on the risk of hypertension disorders in pregnancy.

All genetic instrumental variables related to obesity and HDP were shown in Table 1. Totally, 14 genetic instruments, which located in four genes (POC5, TRIM66, MAP2K5, and FTO) and 12 chromosomes (1, 2, 3, 4, 5, 6, 11, 12, 15, 16, 18, and 19), were included in the present MR analysis. The effect allele frequency (EAF) of SNPs in obesity ranged from 0.1 to 0.9 and the EAF of SNPs in HDP ranged from 0.2 to 0.9. The risk of each genetic variant associations with obesity and HDP is presented in Figures 2, 3.

### MR for HDP

**Figure 4:**
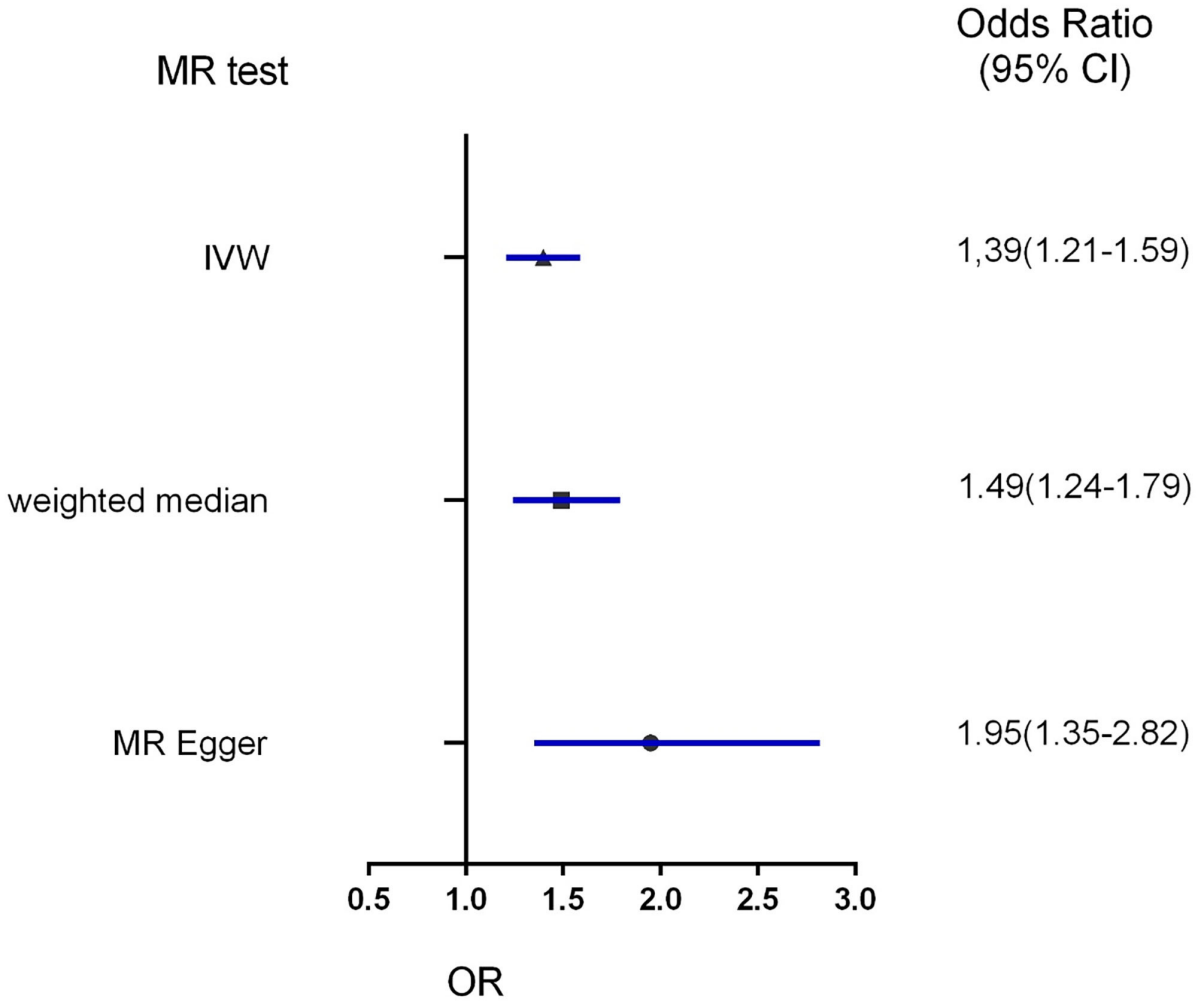
Forest plot to visualize the causal effect of obesity on the risk of hypertension disorders in pregnancy by three methods. IVW indicates inverse-variance weighted; and MR, Mendelian randomization.

Three MR methods including IVW, MR-Egger, and weighted median regression, were used to investigate the causal effects of obesity on the risk of HDP (Figures 2, 4). IVW method showed that genetically predicted obesity was causally associated with a higher risk of HDP [IVW odds ratio (OR) = 1.39; 95%CI, 1.21–1.59); *P* = 2.46 ×10^−6^]. Besides, the MR power is 88% in the present study by using the “mRnd” tool. Weighted median and MR Egger regression also presented similar directed estimates [weighted median OR = 1.49 (95% CI, 1.24–1.79), *P* = 2.45 × 10^−5^; MR-Egger OR = 1.95 (95% CI, 1.35–2.82), *P* = 3.84 × 10^−3^]. The consistency in the results of all three MR approaches suggested that genetically predicted obesity causally reliably increased the risk of HDP.

### Horizontal Pleiotropy Analysis

**Figure 5:**
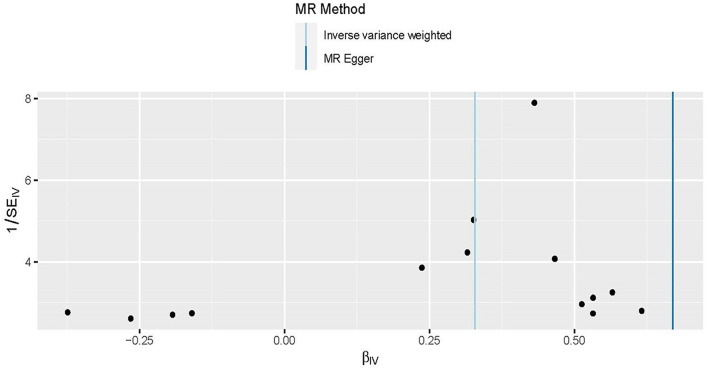
Funnel plots to visualize overall heterogeneity of Mendelian randomization (MR) estimates for the effect of obesity on the risk of HDP. The black point representing an SNP is plotted in relation to the effect size of the SNP on hypertension disorders in pregnancy (x-axis) and reciprocal of the standard errors (y-axis) in the inverse-variance weighted Mendelian randomization. The asymmetry of funnel plots represents directional horizontal pleiotropy, with individual Wald ratios of each SNP that plotted according to its precision.

The asymmetry of funnel plots with individual Wald ratios of each SNP that plotted according to its precision represents directional horizontal pleiotropy. However, it is difficult to investigate the symmetry of a funnel plot when using a small number of IVs (Figure 5). Therefore, the MR-Egger intercept was used to further explore the directional horizontal pleiotropy. And no evidence of directional pleiotropy was shown between obesity and HDP in the present MR analysis (*P* = 0.07).

### The Effects of Individual Genetic Instruments Correlated With HDP

**Figure 6:**
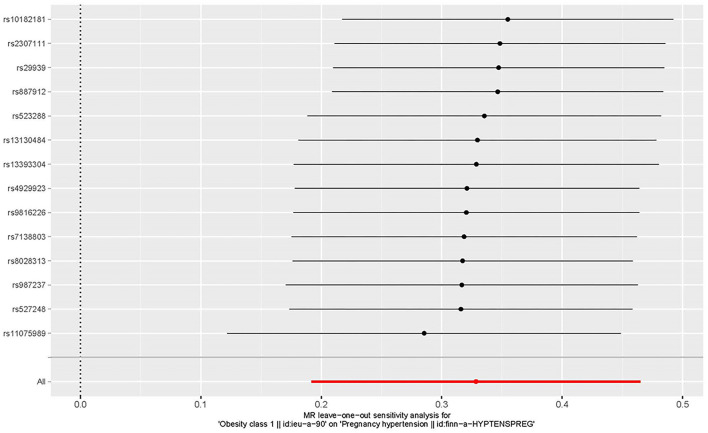
The leave-one-out plot to visualize the causal effect of obesity on the risk of hypertension disorders in pregnancy when leaving one SNP out.

The leave-one-out approach was selected for a sensitivity analysis to confirm the effect of each SNP on the overall causal estimate. When individual SNP was systematically removed and MR analyses were repeated. No substantial difference was observed in estimated causality and the findings have important credibility (Figure 6). Consequently, the estimated effects couldn't be interpreted by any single genetic instrument.

## Discussion

In this two-sample MR study, we firstly assessed the causal association between obesity and HDP based on the European ancestry population. Our findings suggested a causal association between genetically determined obesity and the increased risk of HDP, with an increase in obesity by 1 SD resulting in a 39% increased risk of HDP, which was consistent with previous observational studies (5, 6).

Compared with MR analysis, reverse causation or confounding factors are more susceptible to bias the results of traditionally observational epidemiological studies. MR study relies on a natural random allocation of genetic variation during meiosis, resulting in a random distribution of genetic variation in populations. Since obesity-associated genetic variants are randomly assigned at birth, which occurred before the onset of HDP. Therefore, MR analysis may provide the best evidence for assessing the causal association between obesity and HDP. Our result suggests that obesity itself can causally increase the risk of HDP, which can be interpreted from the genetic perspective in our MR analysis. The result of this MR was consistent with the findings of previous studies, supporting obesity as a risk predictor for HDP (5, 6). We used three different methods to estimate the causal association to minimize potential pleiotropy in the current sensitivity analysis, including IVW, MR-Egger, and weighted median regression. IVW analysis provided obesity as a risk factor for HDP with an OR of 1.39 (95% CI, 1.21–1.59; *P* = 2.46 × 10^−6^). Weighted median and MR Egger regression also showed directionally similar results [weighted median OR = 1.49 (95% CI, 1.24–1.79), *P* = 2.45 × 10^−5^; MR-Egger OR = 1.95 (95% CI, 1.35–2.82), *P* = 3.84 × 10^−3^]. In our MR analysis, all three methods give similar statistically significant results to confirm the reliability of our causal association between obesity and HDP. Previous observational studies have shown obesity is correlated with an increased risk of gestational hypertension, cesarean section rates, and the risk of anesthesia. These emphasize the potential benefit of weight loss among obese women of childbearing age (6). Hence, our results highlight the importance of weight control in pregnant women and provide genetic evidence for future elucidating the pathogenesis of hypertension formation during pregnancy. Gene analyses of lifestyle and environment interaction have suggested that our increasingly obesogenic environment may increase the genetic risk of obesity, but those risks could be reduced by increasing physical activity and avoiding specific dietary components. Exploring a causal association of obesity with HDP, which could be preventable through weight-loss interventions has clinical significance. In other words, verifying the causal detrimental impact between obesity and HDP risk has a great social significance and clinical value.

It has been reported that several genes significantly associated with the increase of obesity risk, which also played an important role in the appetite regulation of hypothalamus (19). rs987237 located in the chromosome 6 transcription factor AP-2 beta gene (*TFAP2B*) has been related to obesity defined by body mass index (BMI), and waist circumference (20), and has been shown interaction with the dietary fat-to-carbohydrate ratio, which has an impact on weight loss. The transcription factor encoded by *TFAP2B* is mainly expressed in adipose tissue, where its regulation of adipocyte function and expression of adipokine is considered to be the functional link to obesity (21, 22), to provide a mechanistic basis for the genetic correlation of *TFAP2B* and obesity. HDP is influenced by environmental factors and a variety of genetics which is a complex disease (23). Epidemiological research has recently examined the correlation between a history of HDP and future risks of other diseases. These studies have reported associations between HDP history and risk of stroke, coronary heart disease, diabetes, heart failure, hypertension, and dysrhythmia. Therefore, reducing the risk of HDP contributes to reducing the risk of diseases for pregnant women. Observational studies have shown a correlation between obesity and HDP, but it is unclear whether the observed associations are causal or caused by confounding bias or reverse causation. Besides, no genetic variants correlated with obesity were reported in HDP, suggesting that the causal correlation between obesity and HDP is independent of the known genetic variants. The most important implication is through public health interventions to reduce the incidence of obesity in pregnant women can make the incidence of high blood pressure in pregnant women during pregnancy lower.

This study has many advantages. We used a two-sample MR design, which minimized confounding and reverse causal bias, to assess the causal association between obesity and increased risk of HDP. To the best of our knowledge, this study is the first MR analysis to investigate the causal association between obesity and the risk of HDP. Besides, several key measures were implemented to meet the basic assumptions of two-sample MR analysis: (1) We only included the SNPs which were correlated with obesity at a GWAS significant level to guarantee an effective correlation between SNPs and risk factors (obesity) in the present MR; (2) All the included GWAS data was finished in the ancestral populations of Europe to reduce the impact of race/ethnicity. Thus, the potential confounding factor is small in this study; (3) MR Egger and weighted median were conducted for the sensitive analysis to guarantee the reliability of our analysis. This study has important clinical implications for predicting HDP risk in clinical practice, which will help rationalize weight-loss strategies to reduce the risk of HDP.

However, the study still had some limitations. First, these data adopted to infer the correlation between obesity and HDP risk factors came mainly from European ancestry, therefore, subsequent studies need to test other regions and races to determine whether the correlation is consistent in other populations (24). Second, we are not able to further explore subgroup analysis on the interest covariates because we just adopted the summary data rather than individual patient data (25).

## Conclusion

In summary, after adopting a two-sample MR analysis, we found a causal correlation between obesity and hypertension disorders in pregnancy. Taking measures to reduce the proportion of obesity may help reduce the incidence of hypertension disorders in pregnancy.

## Data Availability Statement

The original contributions presented in the study are included in the article, further inquiries can be directed to the corresponding authors.

## Author Contributions

WW and J-ST designed the study and wrote the manuscript. WW, J-ST, LH, SZ, and HL contributed to the data analysis and data interpretation. YW and JL contributed to the revision of the manuscript. All authors contributed to the article and approved the submitted version.

## Funding

This work was supported by the National Natural Science Foundation of China Grant Awards (Grant No. 81960669), CAMS Innovation Fund for Medical Sciences (CIFMS) (Grant Nos. 2020-12M-C&T-B-063), Hainan Province Clinical Medical Center, the National Clinical Research Center for Cardiovascular Diseases, Fuwai Hospital, Chinese Academy of Medical Sciences (NCRC2020007), and the Research Project of Clinical Toxicology from the Chinese Society of Toxicology (CST2020CT303).

## Conflict of Interest

The authors declare that the research was conducted in the absence of any commercial or financial relationships that could be construed as a potential conflict of interest.

## Publisher's Note

All claims expressed in this article are solely those of the authors and do not necessarily represent those of their affiliated organizations, or those of the publisher, the editors and the reviewers. Any product that may be evaluated in this article, or claim that may be made by its manufacturer, is not guaranteed or endorsed by the publisher.
